# Supplementary figures and images for: Regulation of hippocampal mossy fiber-CA3 synapse function by a Bcl11b/C1ql2/Nrxn3(25b+) pathway
Source: eLife. 2024 Feb 15;12:RP89854. doi: 10.7554/eLife.89854 (PMC10942602; doi:10.7554/eLife.89854)

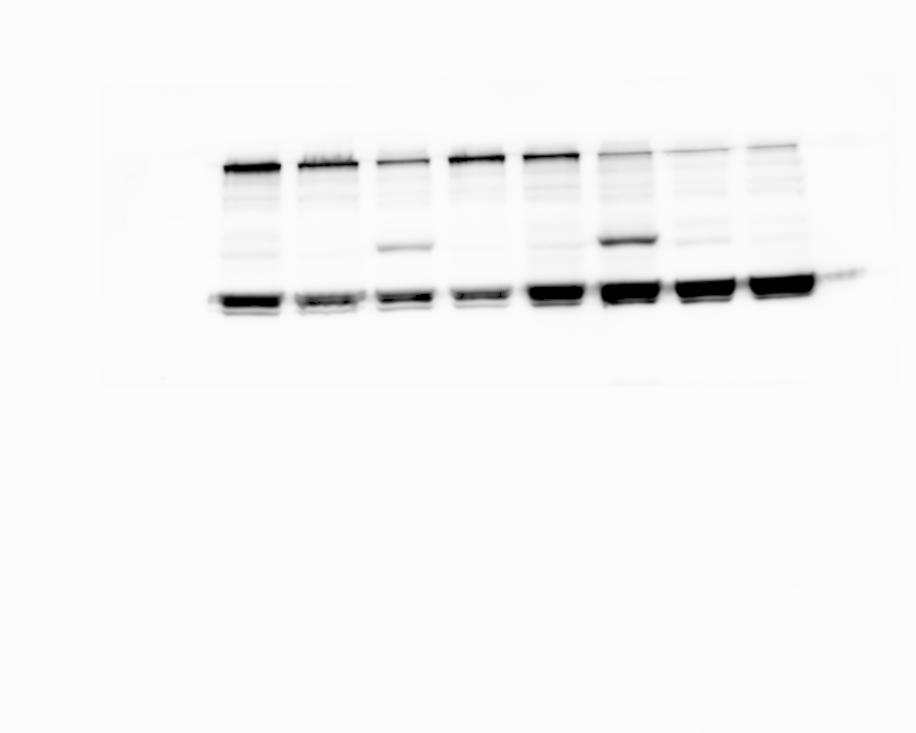

Supplement: Figure 1—source data 2. [file elife-89854-fig1-data2.zip › Koumoundourou et al 2023_Figure1c_Source data 2.tif]

**h**

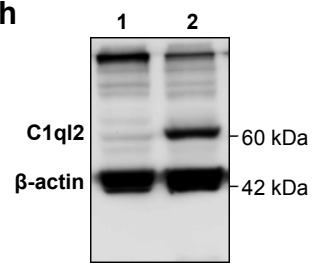

1: Control+EGFP  
2: Control+EGFP-2A-C1ql2

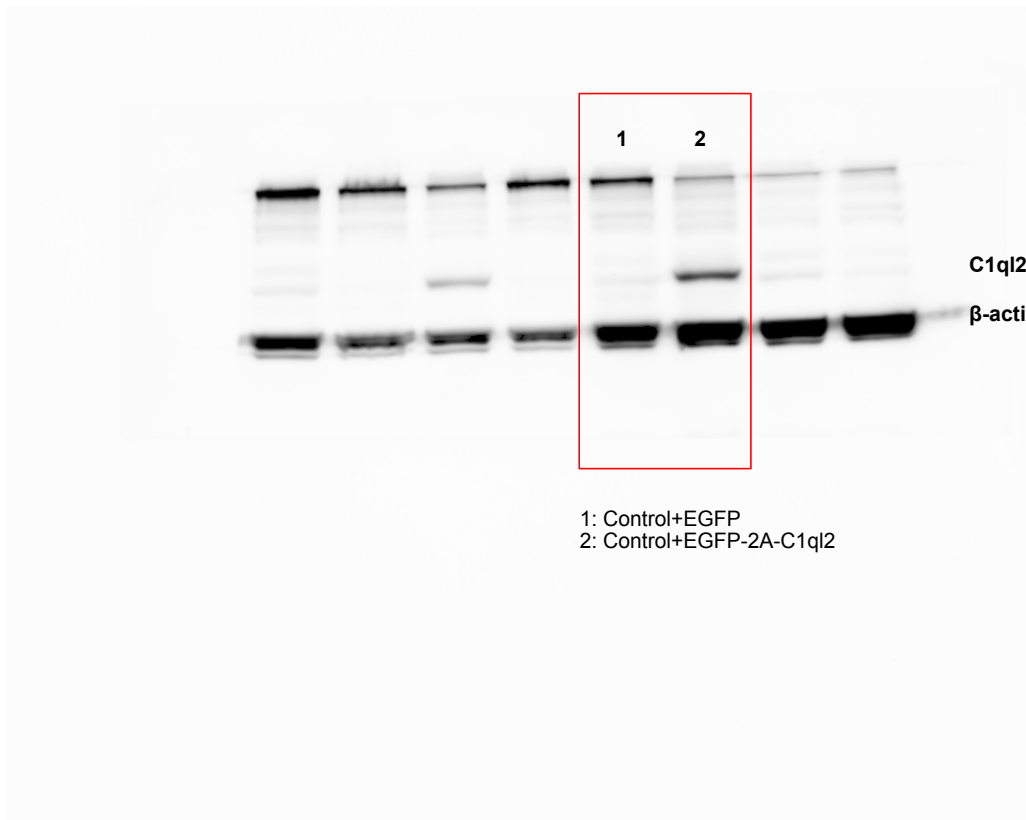

1: Control+EGFP  
2: Control+EGFP-2A-C1ql2

Supplement: Figure 2—source data 3. [file elife-89854-fig2-data3.zip › Koumoundourou et al 2023_Figure 2_ Source data 3.pdf]

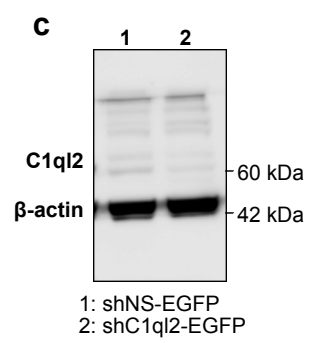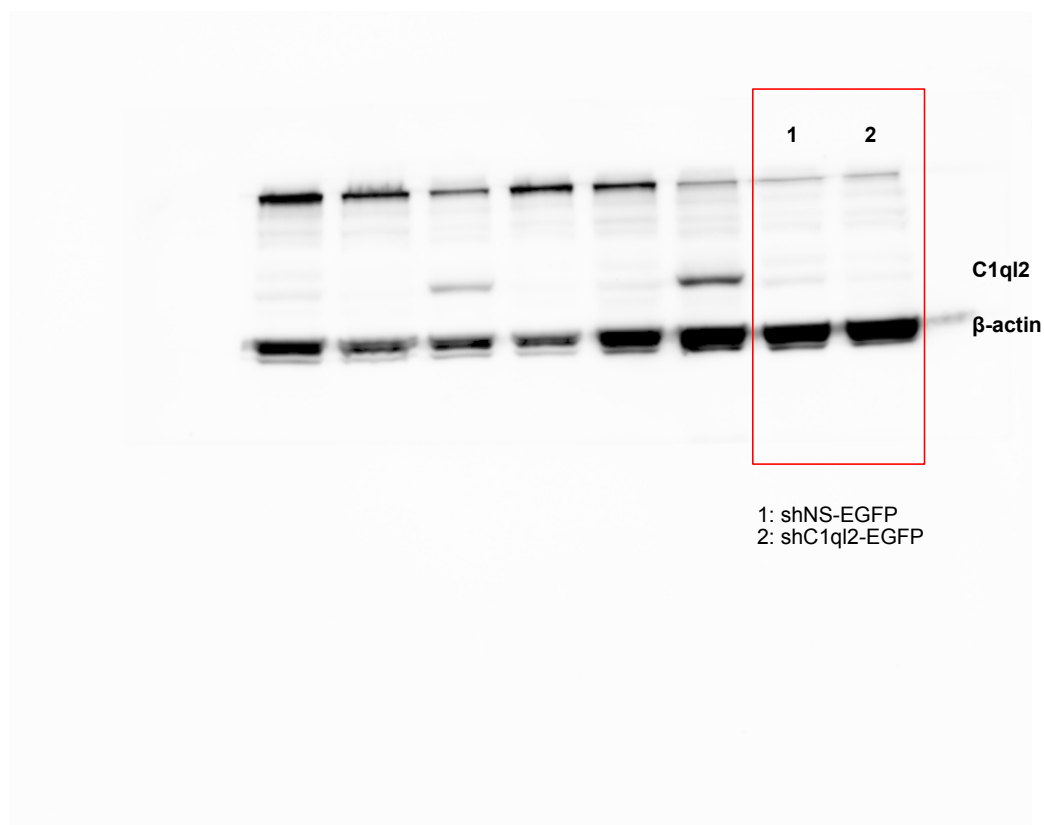

Supplement: Figure 4—source data 3. [file elife-89854-fig4-data3.zip › Koumoundourou et al 2023_Figure 4_ Source data 3.pdf]

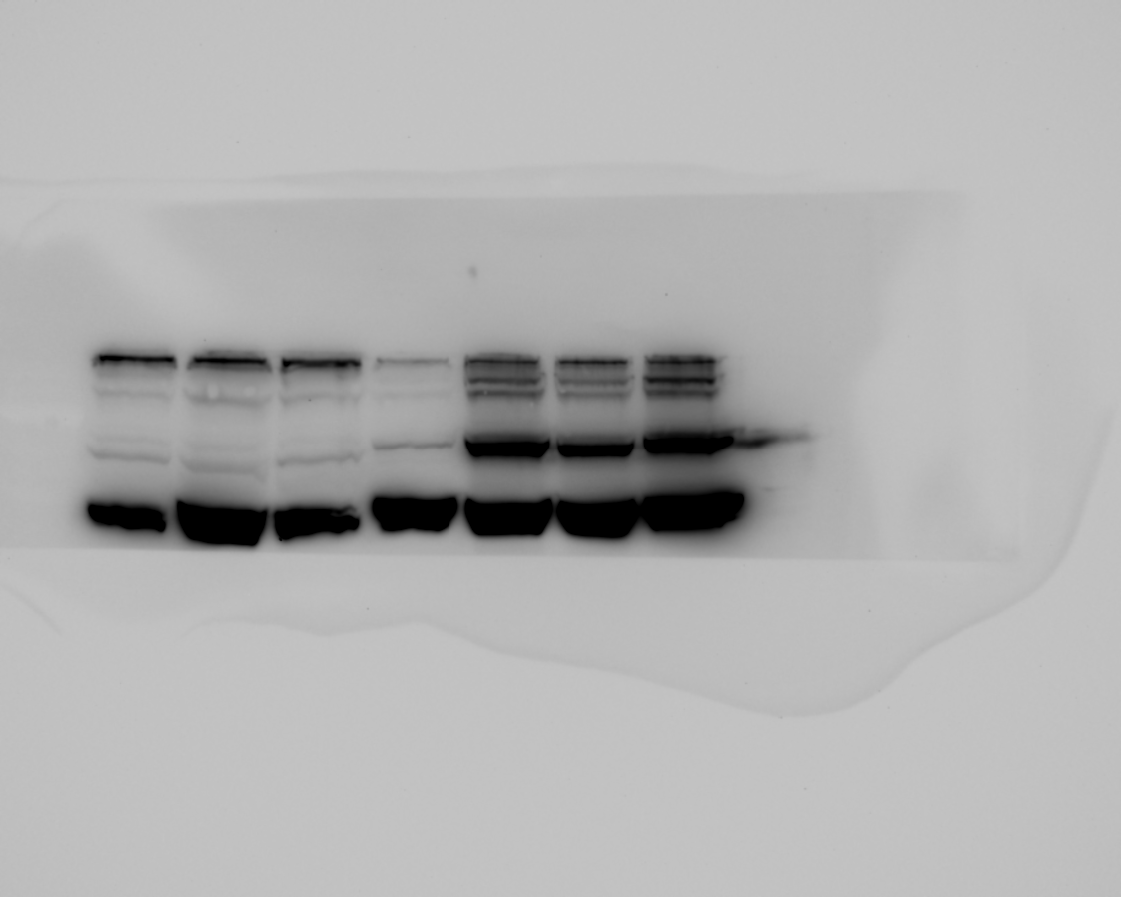

Supplement: Figure 6—source data 2. [file elife-89854-fig6-data2.zip › Koumoundourou et al 2023_Figure 6_Source data 2.tif]

**b**

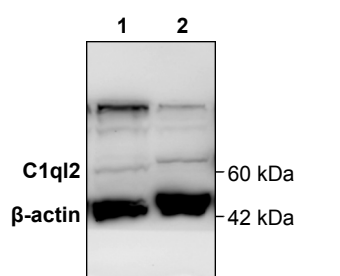

1: Control+EGFP  
2: Bcl11b cKO+EGFP-2A-K262E

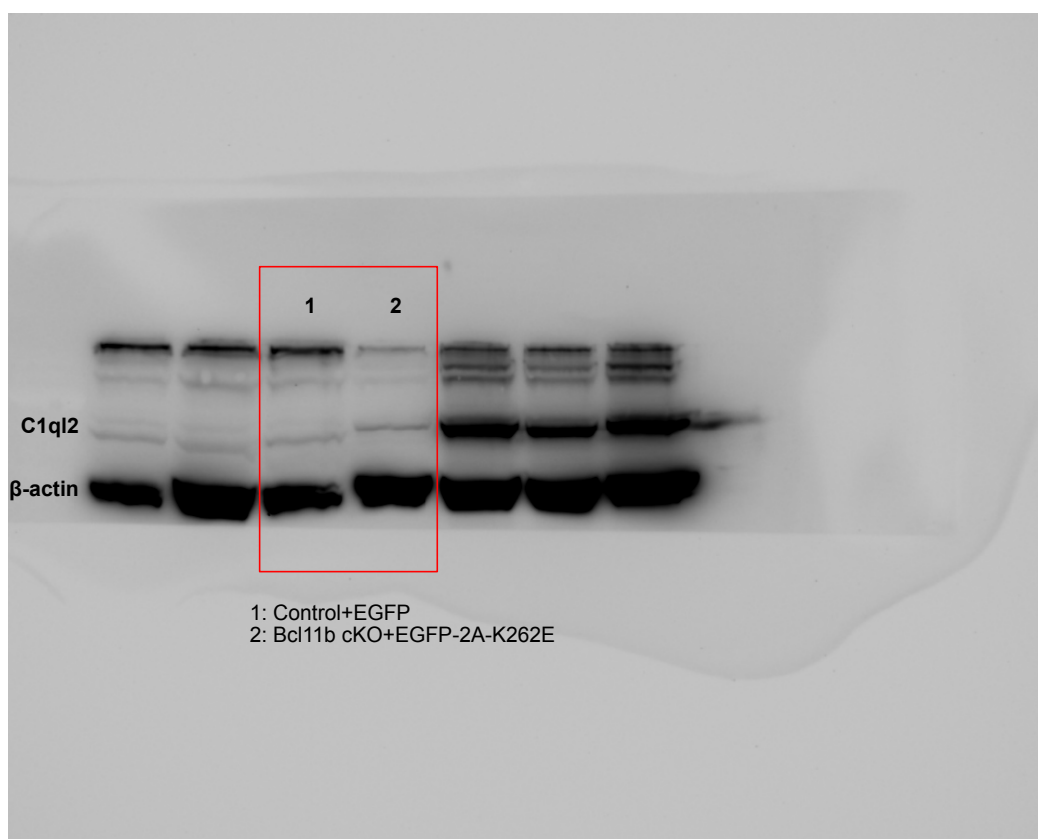

Supplement: Figure 6—source data 3. — Figure 6—figure supplement 1—source data 1. Original file for the western blot analysis in Figure 6—figure supplement 1k. [file elife-89854-fig6-data3.zip › Koumoundourou et al 2023_Figure 6_Source data 3.pdf]

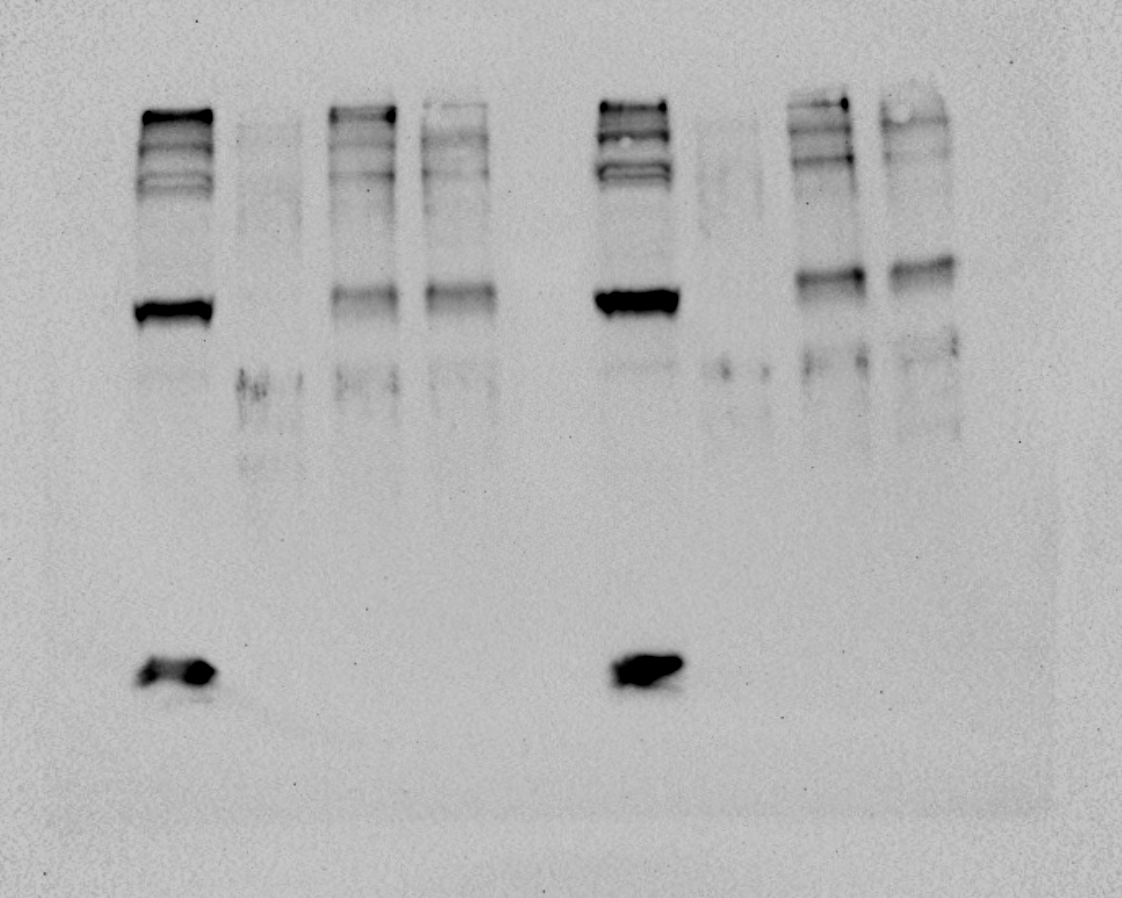

Supplement: Figure 6—figure supplement 1—source data 1. [file elife-89854-fig6-figsupp1-data1.zip › Koumoundourou et al 2023_Figure 6_Supplement figure 1-Source data 1.tif]

**k**

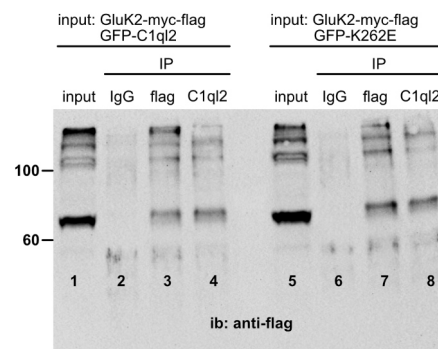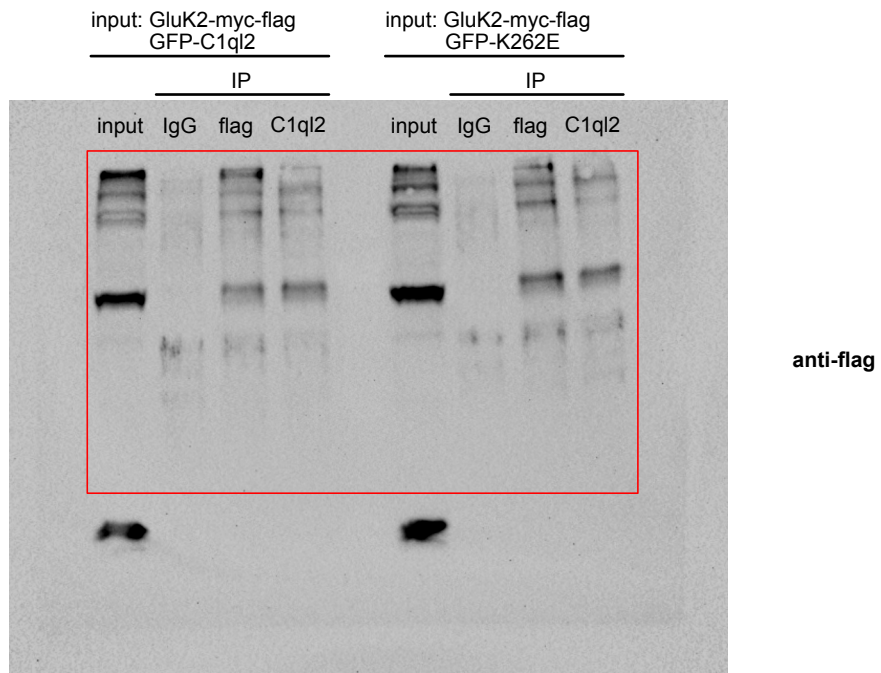

Supplement: Figure 6—figure supplement 1—source data 2. [file elife-89854-fig6-figsupp1-data2.zip › Koumoundourou et al 2023_Figure 6-Supplement figure 1_Source data 2.pdf]
