# Supplementary material for: Regulation of hippocampal mossy fiber-CA3 synapse function by a Bcl11b/C1ql2/Nrxn3(25b+) pathway
Source: eLife. 2024 Feb 15;12:RP89854. doi: 10.7554/eLife.89854 (PMC10942602; doi:10.7554/eLife.89854)
Supplement: Figure 1—source data 3. [file elife-89854-fig1-data3.zip › Koumoundourou et al 2023_Figure 1_Source data 3.pdf]

c

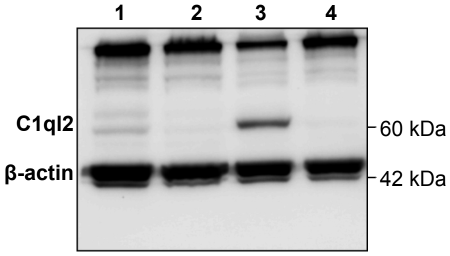

1: Control+EGFP
2: Bcl11b cKO+EGFP
3: Bcl11b cKO+EGFP-2A-C1ql2
4: Bcl11b cKO+EGFP-2A-C1ql3

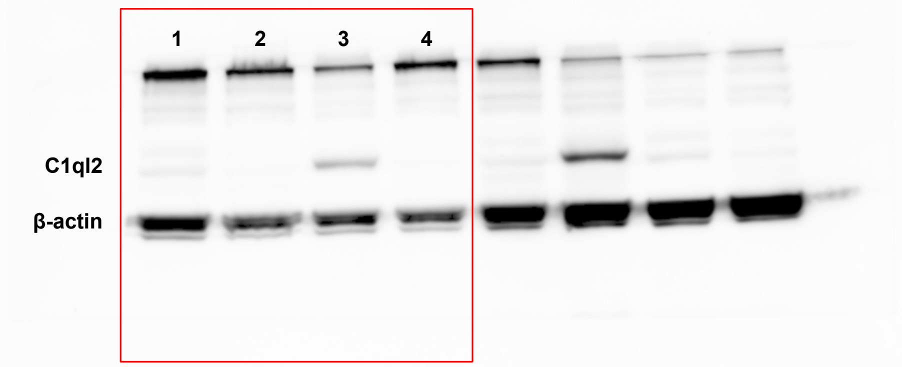

1: Control+EGFP
2: Bcl11b cKO+EGFP
3: Bcl11b cKO+EGFP-2A-C1ql2
4: Bcl11b cKO+EGFP-2A-C1ql3
